# Drug Toxicity Deaths after Release from Incarceration in Ontario, 2006-2013: Review of Coroner’s Cases

**DOI:** 10.1371/journal.pone.0157512

**Published:** 2016-07-06

**Authors:** Emily Groot, Fiona G. Kouyoumdjian, Lori Kiefer, Parvaz Madadi, Jeremy Gross, Brittany Prevost, Reuven Jhirad, Dirk Huyer, Victoria Snowdon, Navindra Persaud

**Affiliations:** 1 Department of Family Medicine, Queen’s University, Kingston, Ontario, Canada; 2 St Michael’s Hospital, Toronto, Ontario, Canada; 3 Ontario Ministry of Community Safety and Correctional Services, Toronto, Ontario, Canada; 4 Dalla Lana School of Public Health, Unviersity of Toronto, Toronto, Ontario, Canada; 5 Centre of Forensic Sciences, Toronto, Ontario, Canada; 6 Faculty of Medicine, University of Toronto, Toronto, Ontario, Canada; 7 Office of the Chief Coroner for Ontario, Toronto, Ontario, Canada; 8 St Michael’s Hospital, Toronto, Ontario, Canada; 9 University of Toronto, Toronto, Ontario, Canada; Medical University of Vienna, AUSTRIA

## Abstract

**Background:**

There is an increased risk of death due to drug toxicity after release from incarceration. The purpose of this study was to describe the timing, rate and circumstances of drug toxicity deaths following release from incarceration. This information can be used to help design potential preventive interventions.

**Methods and Findings:**

We reviewed coroner’s files to identify deaths in adults in Ontario between 2006 and 2013 caused by drug toxicity (n = 6,978) and these records were matched with provincial correctional records to identify individuals who died within one year of being released from incarceration (n = 702). Twenty percent (n = 137) of the 702 deaths occurred within one week of release. The majority (77%, n = 538) of deaths after release involved one or more opioids. Of the deaths involving opioids, intervention by another person may have been possible in 318 cases.

**Conclusions:**

Between 2006 and 2013 in Ontario, one in ten drug toxicity deaths in adults occurred within one year of release from provincial incarceration. These findings may help to inform the implemention and assessment of interventions aimed at reducing drug toxicity deaths following release from incarceration.

## Introduction

An increased risk of all-cause mortality following release from incarceration has been documented in Australia [1,2], Finland [3,4], France [5], Scotland [6], Canada [7], and the United States [8–10]. Drug toxicity,the adverse effects of both therapeutically used and misused substances, is a major contributor to the increased mortality risk [6–9,11–13]. Drug-related standardized mortality ratios (SMRs) for ex-prisoners compared to reference populations range from 2.2 to 274.2 [14]. The risk of death due to drug toxicity is especially high during the first two weeks after release [6–9,11], likely because imprisonment can reduce drug use and may result in loss of drug tolerance [8,12,13,15]. Interviews with ex-prisoners in the United States revealed that often they were released into environments where drug use was pervasive and that various stressors contributed to drug use [16]. Although most post-release deaths due to drug toxicity occur in men because of their higher rate of incarceration [6,8,9,11], the drug toxicity mortality rate post-release is higher in women than in men [9]. Opioids are the most common drug resulting in death in individuals recently released from incarceration [9].

Potential interventions to prevent toxicity deaths include abstinence from drug use, reducing drug use, safer prescribing, harm reduction strategies that promote safer drug use, substitution therapies, education about how to respond appropriately to a potential substance toxicity, policy that promotes use of emergency services when drug toxicitiy is suspected, and access to reversal agents such as naloxone for opioid toxicity [17–20]. The purpose of this study was to describe the timing, rate, and circumstances of drug toxicity deaths following release from incarceration. This information can be used to help design potential preventive interventions including, but not limited to, naloxone programs.

## Methods

This is a descriptive retrospective longitudinal study linking records of drug toxicity deaths from the Office of the Chief Coroner of Ontario to incarceration records from Correctional Services, both part of the Ontario Ministry of Community Safety and Correctional Services. In Ontario, all sudden and unexpected deaths are reportable to a coroner and this includes deaths due to accident (unintentional events, including overdose), homicide, suicide, or unknown causes [21].

The Ontario Ministry of Community Safety and Correctional Services operates all provincial correctional facilities for adults in Ontario. Ontario’s provincial correctional facilities house unsentenced persons who are detained before and during trial, those who are serving sentences of less than two years, and some individuals being detained for certain immigration-related issues; throughout this article, we refer to these groups as “incarcerated.” Approximately 50,000 individuals are released from provincial incarceration annually. Individuals sentenced to two years or more are usually incarcerated in federal penitentiaries. This study population is limited to individuals 18 years or older who were released from a provincial correctional facility within the year prior to death.

All drug toxicity deaths that occurred between 2006 and 2013 (inclusive) in Ontario that were investigated by a coroner were identified by a keyword search of the Coroner’s Investigation System. The resulting list of names, dates of birth, and dates of death was provided to Correctional Services to identify all individuals from this list who had been incarcerated in a provincial facility in Ontario in the year preceding death by exactly matching names and dates of birth. For all matched individuals, Correctional Services provided the start and end dates of all incarcerations.

We reviewed coroner’s files, which each contain a coroner’s investigation statement and which may contain a police report, autopsy report, toxicology report, medical record, and ambulance report. Six data abstracters (BP, DH, EG, JG, TN, VS) used a standardized data extraction tool that was piloted prior to use to systematically extract from the file: demographic information, the cause and immediate circumstances of death, toxicology findings, autopsy findings, medical history, and whether or not intervention prior to death by another individual was possible. Natural deaths were excluded as this is a study of drug toxicity deaths. Deaths that occurred in provincial correctional facilities were excluded, but deaths that occurred in police custody prior to transfer to a provincial correctional facility were included. Three reviewers (EG, NP, VS) discussed each case to determine whether intervention by another person was possible using the rules outlined in S1 Appendix. Briefly, intervention was deemed possible if a person was not alone around the time of his or her death or if others were aware that a person was intoxicated prior to his or her death. For cases where consensus could not be reached, the majority opinion was used.

To assess the reproducibility of the data extraction process, 50 completed case files were randomly selected and information from these files was independently extracted by two data extractors (EG and VS) who were blinded to the original extraction results. There were no important discrepencies.

We calculated descriptive statistics for age, sex, manner of death, incarceration type and length, and substance type. The expected number of deaths was calculated using all drug toxicity deaths in individuals 18 years or older identified by the keyword search of the Coroner’s Investigation System database as well as the mean age- and sex-specific Ontario population over the study period [22]. Four cases with erroneous release dates were excluded from figures involving release dates. The annual number of individuals released from provincial incarceration between fiscal years 2005–6 to 2013–14 was used for the calculation of the mortality rates. Microsoft Excel 2010 was used for all statistical analyses [23].

Ethics approval for this study was received from the University of Toronto Research Ethics Board (2014–007). The study was also approved by the Ontario Ministry of Community Safety and Correctional Services Research Committee. Consent was not sought as the study includes only previously collected information regarding deceased individuals and this decision was approved by both the University of Toronto Research Ethics Board and the Ontario Ministry of Community Safety and Correctional Services Research Committee. No identifying data were used in the analyses.

## Results

**Fig 1:**
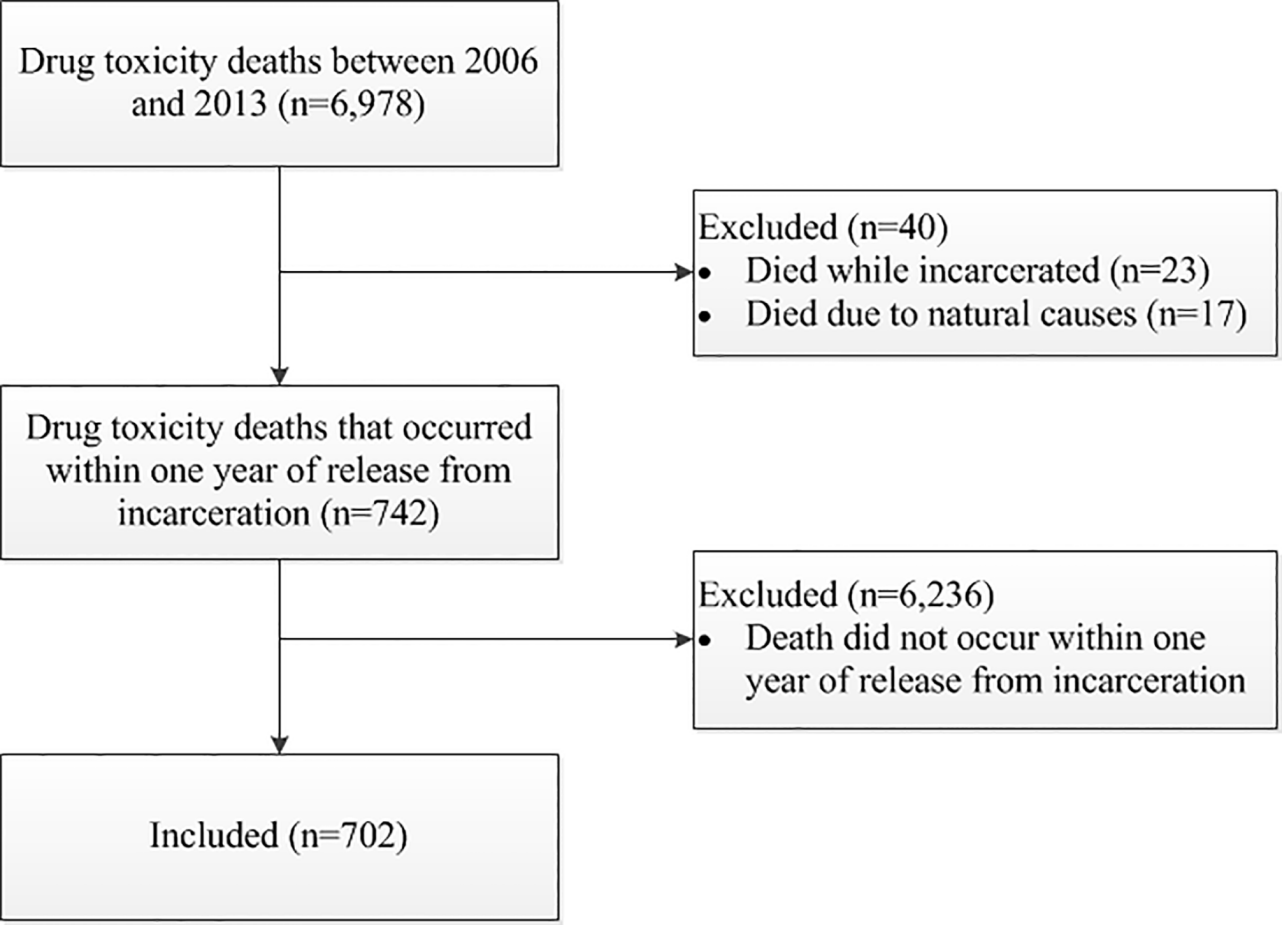
Flow diagram of study participants.

We identified 6,978 deaths due to drug toxicity in Ontario between 2006 and 2013. Of the drug toxicity deaths, 742 exactly matched records of individuals released from provincial incarceration in the year prior to death. Of the matched cases, 23 died while incarcerated and 17 died of natural causes, so were excluded from the study sample. The 702 remaining persons were included in the study (Fig 1).

Most decedents were men younger than 55 years of age who died of accidental (unintentional) causes of deaths after being released from remand (Table 1). The distribution of incarceration length was right-skewed, with a median length of incarceration of 25 days and a mean of 62 days.

**Table 1:** Characteristics of persons who died due to drug intoxication in the year after release from incarceration in Ontario, 2006–2013, n = 702.

Characteristic		Number (percent of total)
Total		702 (100)
Age at death	18–24 years	77 (11)
	25–34 years	207 (30)
	35–44 years	232 (33)
	45–54 years	156 (22)
	55–64 years	27 (3.8)
	≥65 years	3 (0.4)
Sex	Male	589 (84)
	Female	113 (16)
Manner of death	Accident (unintentional)	585 (83)
	Suicide	56 (8.0)
	Undetermined	61 (8.7)
	Homicide	1 (0.1)
Incarceration type	Remanded (detained prior to or during a criminal proceedings)	585 (83)
	Sentenced	58 (8.2)
	Other (immigration detentions, national parole violation, police custody)	59 (8.4)

Compared to the general population, the standardized mortality ratio (SMR) in the year after release from custody was 11.59 (95% CI, 6.38–16.79). Table 2 shows the expected and observed deaths by age group used to calculate the SMR.

**Table 2:** Expected and observed mean annual deaths for persons who died due to drug intoxication in the year after release from incarceration in Ontario, 2006–2013.

	Men		Women	
Age at death (years)	Expected mean annual deaths	Observed mean annual deaths	Expected mean annual deaths	Observed mean annual deaths
All	6.9	72	0.52	14
20–24	0.69	6.6	0.041	1.5
25–29	1.2	10	0.071	2.8
30–34	1.1	11	0.073	2.4
35–39	1.0	10	0.081	1.5
40–44	1.2	15	0.10	2.4
45–49	0.97	9.2	0.085	2.4
50–54	0.54	7.1	0.045	0.75
55–59	0.17	2.6	0.013	0.25
60–64	0.048	0.50	0.004	0
65–69	0.012	0.25	0.001	0
70–74	0.006	0.12	0	0

Ontario population data from reference 22

**Fig 2:**
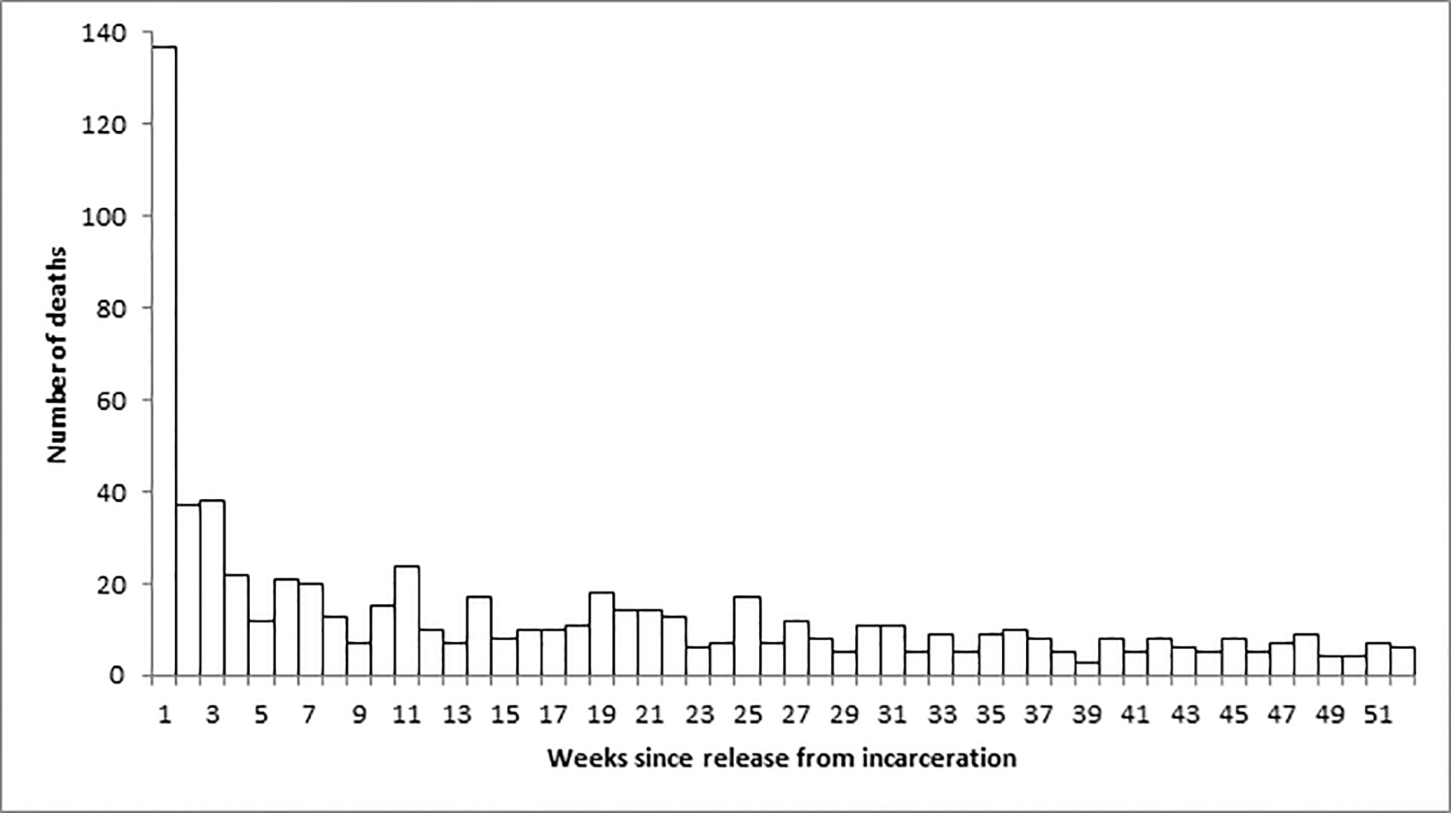
Number of deaths by week since release in the year after release from provincial incarceration, 2006–2013.

**Fig 3:**
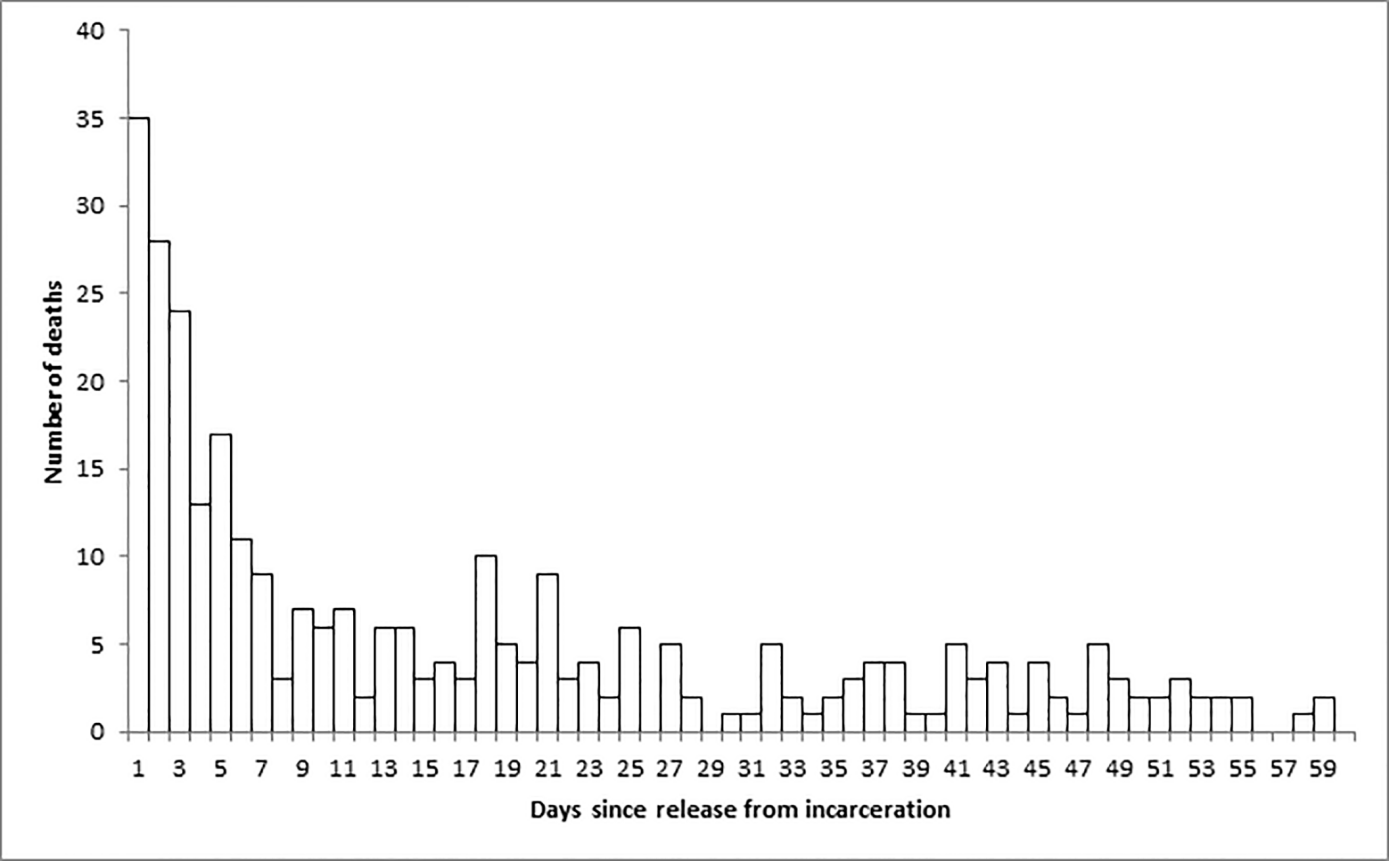
Number of deaths by day since release in the year after release from provincial incarceration, up to day 60, 2006–2013.

Nine percent of deaths (n = 63) occurred within two days of release from incarceration and 20 percent of deaths (n = 137) occurred within one week of release (Figs 2 and 3). While the death rate was highest immediately after release, the majority of deaths occurred more than a month after release; the median length of time between release and death was 78 days, while the mean was 113 days.

Just over half of the deaths were associated with mixed substance toxicity. Of the deaths due to single substance toxicity, more than half were due to opioids. The majority of the deaths (77%, n = 538) were due to opioid toxicity alone or mixed drug toxicity including opioids (Table 3). The most common non-opioid substance causing death was cocaine.

**Table 3:** Substances causing death in persons in the year after release from incarceration in Ontario, 2006–2013, n = 702.

Substance			Number (percent of total)
Mixed substance toxicity*			391 (56)
		Mixed, including at least one opioid	346 (49)
		Mixed, including cocaine	180 (26)
		Mixed, including at least one opioid and cocaine	163 (23)
Single substance toxicity			309 (44)
	Opioids		192 (28)
		Oxycodone	48 (6.8)
		Fentanyl	42 (6.0)
		Methadone	35 (5.0)
		Morphine	30 (4.3)
		Heroin	25 (3.6)
		Hydromorphone	10 (1.4)
		Other opioids	2 (0.2)
	Non-opioids		117 (17)
		Cocaine	73 (10)
		Quetiapine	10 (1.4)
		Methamphetamine	8 (1.1)
		Other non-opioids	26 (3.7)
	Unknown		2 (0.2)

*The sum of the sub-groups is greater than the total because the sub-groups overlap.

In 677 cases (96%), we were able to determine whether another person was present or aware of the decedent’s intoxication. Intervention by another person was deemed possible in 412 (59%) of these cases. Of the deaths involving opioids, intervention by another person was deemed possible in 318 cases (59% of deaths involving opioids, representing 45% of all drug-toxicity deaths).

## Discussion

### Interpretation

Annually in Ontario, approximately 88 individuals die due to drug toxicity in the year after release from provincial incarceration, corresponding to one in ten of all drug toxicity deaths in adults in Ontario. The death rate is highest in the weeks following release. Most deaths involve opioids and often another person who could have intervened was present.

The increased rate of death immediately following release is consistent with previously published literature [12,24,25], and is thought to be partly due to the use of drugs after a period of abstinence that had resulted in loss of drug tolerance [8,12,13,15]. Drug use is reduced, but may not be entirely prevented, during imprisonment. In a study of federal prisoners in Canada, 22 percent of men reported using injection drugs prior to incarceration, and 17 percent of men reported using injection drugs while incarcerated [26]. A recent survey of men in an Ontario provincial detention centre found that more than half reported using illicit drugs other than cannabis in the year prior to incarceration, 22% of whom reported injecting drugs [27]. The high death rate immediately after release suggests that preventive measures might be most effective if they are started prior to release. Healthcare services are publicly funded for most residents of Ontario including those released from incarceration; during the study period there were no substantial changes in the healthcare of individuals released from incarceration.

Almost 12 times more drug toxicity deaths occur in recently released prisoners than would be expected based on the number of drug toxicity deaths in the general population. This is consistent with previously published SMRs, which reflect that recently released prisoners are at a greater risk of death due to drug toxicity than the general population [14]. Consistent with the sex and age demographics of incarceration in Ontario, the bulk of deaths occur in young men; however, the highest death rates are among womenThe high death rates underscore the particular need for implementation and evaluation of drug toxicity prevention measures tailored to people recently released from incarceration [7,17]

Almost 77 percent of deaths in this study were related to the use of opioids. This is greater than the Ontario average: in a study of all drug-related deaths in Ontario, 58 percent involved opioid use [28]. In other studies of deaths due to drug toxicity in individuals recently released from incarceration, the proportion involving opioids ranges from 58.6 percent in Washington State [9] to 89 percent in Ireland [25] to 95 percent in England and Wales [13]. The large fraction of deaths involving opioids suggests that preventive measures directed at opioids might have the largest impact, such as safer prescribing practices, opioid substitution therapies, overdose prevention education, and opioid reversal agents such as naloxone.

Our findings indicate that it may have been possible for a bystander to intervene in 59 percent of the opioid-related deaths. The World Health Organization strongly recommends that individuals likely to witness opioid overdose should have access to, and be trained to administer, the opioid antagonist naloxone [29]. Following the introduction of the National Naloxone Programme in Scotland, which distributes naloxone kits to prisoners, amongst others, the percentage of opioid-related deaths occurring within four weeks of release from incarceration dropped from 9.8 percent in 2006–2010 to 4.7 percent in 2013, although the total number of drug-related deaths was not affected [20]. Our findings support the ongoing evaluation of naloxone distribution among people recently released from incarceration.

Prison-based methadone maintanence programs have been shown to reduce mortality in the post-release period [30,31]. Opioid substitution therapy using methadone or buprenorphine is available in Ontario provincial prisons, although there may be barriers to access both before and after release [32].

A recent systematic review of randomized controlled trials examining interventions to improve the health of prisoners before and after release identified a lack of evidence on interventions to prevent mortality after release [33]. There is some evidence that modified therapeutic community substance abuse treatment reduces substance use after release [34,35]. Evidence regarding the effectiveness of community re-entry programs and community case management at reducing substance use after release is mixed [36–39]. There is some evidence that the use of motivational interviewing by prison staff and cognitive-behavioural therapy does not reduce substance use after release [40,41]. More evidence on interventions, both during and after incarceration, is required to make informed policy and program decisions to reduce the risk of death due to drug toxicity after release from incarceration.

### Strengths and limitations

All drug toxicity deaths in Ontario are reportable to a coroner, so detailed records of the cause and circumstances of each of these deaths should be available. Unlike the database linkage studies typically used to examine death following incarceration, coroner’s records provide detailed information on the circumstances of death and allowed the determination of whether intervention by another person prior to death was possible.

The major limitation of this study is that these data were collected for administrative use. There were some apparent errors in data entry (e.g., four cases had a negative length of time between release from incarceration and death). Errors in names, aliases, or changes to name spelling or errors in dates of birth may have resulted in an underestimation of the number of deaths. Toxicological testing is sometimes ceased when a potentially fatal concentration of one substance is identified even though there might be other toxicologically significant substances present, which could result in an underestimate of the proportion of deaths in which each substance was involved and an overestimation of the number of deaths involving a single substance. Federal incarceration data were unavailable, and including these data would likely increase the number of deaths in the post-release period. We did not have information about prior substance use of decedents.

## Conclusion

Between 2006 and 2013 in Ontario, one in ten drug toxicity deaths in individuals over the age of 18 occurred within one year of release from provincial incarceration. The death rate is highest immediately after release. These findings may help inform the implementation and evaluation of interventions to reduce drug toxicity deaths following release from incarceration.

## Supporting Information

S1 Appendix Rules used to determine if intervention by another person was possible prior to death. (DOCX)

## Abbreviations

SMR: Standardized mortality ratio
