# Supplementary material for: Drug Toxicity Deaths after Release from Incarceration in Ontario, 2006-2013: Review of Coroner’s Cases
Source: PLoS One. 2016 Jul 6;11(7):e0157512. doi: 10.1371/journal.pone.0157512 (PMC4934911; doi:10.1371/journal.pone.0157512)
Supplement: S1 Appendix — (DOCX) [file pone.0157512.s001.docx]

| Determination | Circumstances prior to death |
| --- | --- |
| Intervention by others possible | - Decedent not alone around time of death (including in police custody, in a health care facility, or with another person who was sleeping) - Unable to determine if decedent alone around time of death; others aware decedent was intoxicated (including situations in which the decedent was found alone, but there was evidence that a party had occurred prior to death) - Decedent alone around time of death; others aware decedent was intoxicated - Decedent alone around time of death; no one else aware decedent was intoxicated; others nearby; public space; passersby activated EMS - Decedent alone around time of death; no one else aware decedent was intoxicated; others nearby; not a public space; no reasonable expectation of privacy (e.g., shared common space, shared bedroom) |
| Intervention by others not possible | - Decedent alone around time of death; no one else aware decedent was intoxicated; others nearby; public space; low-traffic or difficult-to-access location - Decedent alone around time of death; no one else aware decedent was intoxicated; others nearby; not a public space; reasonable expectation of privacy (e.g., bathroom, own bedroom) - Decedent alone around time of death; no one else aware decedent was intoxicated; no others nearby |
| Unable to determine if intervention by others possible | - Unable to determine if decedent alone around time of death; no one else aware decedent was intoxicated - Decedent alone around time of death; no one else aware decedent was intoxicated; others nearby; public space; unable to determine if passersby aware decedent required intervention |
